# Risk of Environmental Chemicals on Bone Fractures Is Independent of Low Bone Mass in US Adults: Insights from 2017 to 2018 NHANES

**DOI:** 10.3390/metabo13030346

**Published:** 2023-02-26

**Authors:** Run Ling, Yuanli Ai, Chengzhi Chen, Jun Zhang, Zhen Zou, Shuqun Cheng, Chunli Li, Xi Li, Bin Wang

**Affiliations:** 1 Institute of Life Sciences, Chongqing Medical University, Chongqing 400016, China; 2 Department of Occupational and Environmental Health, College of Public Health, Chongqing Medical University, Chongqing 400016, China

**Keywords:** environmental chemical factors, bone mineral density, osteopenia, bone fracture, American adults, National Health and Nutrition Survey

## Abstract

To assess the association of environmental chemical factors with osteopenia and/or bone fractures. All data were extracted from the National Health and Nutrition Survey (NHANES) 2017–2018 of American adults aged 20–59 years old; invalid data were excluded based on dual-energy X-ray absorptiometry. For the ultimate valid data set, multivariate logistic regression models were applied to evaluate the association of environmental chemical factors with osteopenia and bone fractures. The valid dataset was obtained from 2640 individuals, who completed a questionnaire of demographic characteristics. Urinary manganese and monomethylarsonic acid were positively associated with osteopenia in American adults, but not bone fracture. However, several environmental factors (e.g., arsenous acid, arsenocholine, dimethylarsinic acid, and 2-thioxothiazolidine-4-carboxylic acid) did not affect bone mineral density, but were significantly associated with bone fracture. Multiple environmental chemical factors significantly affect bone mass or fracture risk. However, the risk of environmental chemical factors on fractures is independent of osteopenia in US Adults. The influence of environmental chemical factors on bone quality should be considered and monitored.

## 1. Introduction

Osteoporosis is a chronic skeletal disorder, characterized by compromised bone mineral density (BMD) and bone strength, impaired bone microstructure, and increased fracture risk at several sites (e.g., hip, wrist, spine, and other skeletal sites). BMD is the commonly used parameter for bone health evaluation; the total body and lumbar spine are preferred sites for BMD assessment [1]. Bone mass increases significantly during childhood and adolescence, and reaches its peak value at approximately 20 years [2]. Reduced BMD could result in osteoporosis [3]. Numerous risk factors contribute to BMD reduction and bone fracture, including nutritional, genetic, and metabolic factors, low physical activity, drugs, and cigarette smoking [4,5,6]. Additionally, environmental factors play an important role in bone health.

It has been confirmed by some evidence that bone health can be affected by heavy metals. In a retrospective study of itai–itai disease cases, Inaba et al. [7] found that high-dose cadmium exposure induced generalized osteomalacia and osteoporosis, resulting in multiple bone fractures. Increased osteoporosis-related bone fracture risk has also been observed after high selenium level exposure [8]. However, heavy metals are not only detrimental to bone health. Existing studies indicated that zinc and mercury deficiency cause bone loss [9,10]. Campbell et al. [11] observed that higher lead exposure led to higher BMD in children. Furthermore, dietary manganese intake is positively correlated with lumbar BMD. Lower copper levels were associated with lower BMD, and long-term exposure to manganese and copper increased the risk of osteoporosis [12,13,14]. As well as heavy metals, polycyclic aromatic hydrocarbons (PAHs) are toxic to bone health; they are characterized by disrupted bone homeostasis [15], leading to the inflammation of various joints with synovitis, progressive bone deterioration, and bone metabolic diseases [16,17,18].

Most previous studies have focused on the impact of a single environmental factor on BMD or fracture, and believed that reducing bone mineral density would lead to fracture to a certain degree. In this cross-sectional study, we analyzed the effect of different environmental chemical factors on BMD and bone fractures based on data from the 2017–2018 cycle of the National Health and Nutrition Survey (NHANES), and explored the association of multiple environmental chemical factors with BMD and bone fractures.

## 2. Materials and Methods

### 2.1. Data Sources and Study Design

The NHANES is a population-based, national, cross-sectional survey, initiated by the National Health Statistics Center of the US. The database combines information collected in interviews and physical examinations to collect health and nutritional status data of US adults and children from a representative sample of the noninstitutionalized civilian population; data are released every 2 years. In this study, we extracted demographic data from the 2017–2018 cycle of the NHANES, comprising data from 9254 individuals. We screened 3419 individuals in the 20–59 age range, of whom 2253 individuals underwent dual-energy X-ray absorptiometry (DXA) to determine total BMD. According to valid DXA data and self-reported information regarding fractures of the hip, wrist, and spine; in total, data were included for analysis of the associations of environmental chemical factors with BMD and bone fracture from 2640 individuals (aged 20–59 years) and 292 individuals (aged 50–59 years), respectively. For dual-energy x-ray absorptiometry, the inclusion criteria in the present study included the following: (1) participants aged 20–59 years; (2) whole body scan completed; (3) all regions were valid. The exclusion criteria were as follows: (1) participants with a positive urine pregnancy test and/or self-reported pregnancy; (2) a history of radiographic contrast material use in past 7 days; (3) weight over 450 pounds or height over 6′5″ (DXA table limitation). In this study, all data were recorded in the NHANES, which the Ethics Committee of the National Health Statistics Center approved.

### 2.2. Biomonitoring

Urine samples were collected from one-third of eligible subjects in the cohort, processed, stored at −30 °C, and shipped to the US National Center for Environmental Health for testing. The concentrations of a number of metals in urine specimens were determined by inductively coupled plasma–mass spectrometry with dynamic reaction cell technology (ICP-DRC-MS). Ultra-performance liquid chromatography coupled with electrospray tandem mass spectrometry was used to measure volatile organic compound (VOC) metabolites [19,20]. For speciated arsenic, high-performance liquid chromatography was used to separate the species and coupled to ICP-DRC-MS to detect arsenic concentration [21].

### 2.3. Bone Densitometry

The reference range for standardizing different bones is determined according to the International Committee for Standards in Bone Densitometry [22], and classified by gender, race, and ethnicity. Based on the T-score formula, a BMD value between 1 standard deviation (SD) and 2.5 SD below the reference mean indicates osteopenia, while a value over 2.5 SD indicates osteoporosis [23].

In the NHANES, the DXA examination provided representative BMD data for different bone tissue sites, including the whole body, trunk, pelvis, thoracic vertebrae, lumbar vertebrae, and limbs. The examinees in the 2017–2018 NHANES were between 8 and 59 years old. Herein, we selected the adult data from the participants aged 20–59 to analyze, and pregnant women were excluded. In the DXA, the whole body was scanned using a Hologic Discovery Model A densitometer (Hologic, Inc., Bedford, MA, USA), and information was collected using Hologic Apex version 3.2 software. All scans were analyzed using Hologic Apex version 4.0 software [24].

### 2.4. Statistical Analysis

All statistical analyses were conducted using R version 4.2.1 and SPSS version 26.0 for Windows (IBM); *p* < 0.05 was considered statistically significant. A multivariate logistic regression model was applied to evaluate correlations of different environmental chemical factors with BMD and fracture of the hip, wrist, and spine; categorical outcomes were reported through *p* value and the odds ratio with a 95% confidence interval (CI). The multivariate logistic regression model was adjusted to control for potential confounders by covariates, including age, gender, body mass index (BMI), race or ethnicity, education level, country of birth, the ratio of family income to poverty, and current smoking status.

## 3. Results

### 3.1. Demographic Characteristics of Participants with Osteopenia

**Figure 1:**
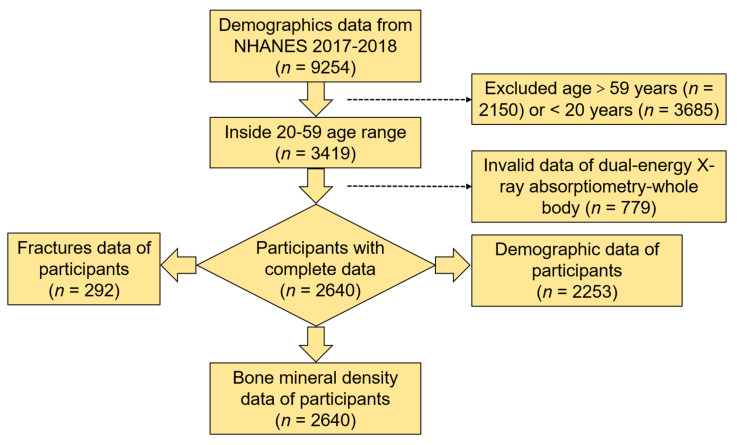
Flow chart of the selection process from the 2017–2018 NHANES.

In this study, 3419 individuals aged 20–59 years (mean, 40.24 years; 95% CI, 39.80–40.69 years) from the 2017–2018 NHANES completed a questionnaire on demographic characteristics. Using the DXA scans as a further selection criterion, the final valid data for analysis was from 2640 individuals. The specific selection process is shown in Figure 1.

Using total BMD as a selection criterion, 2253 individuals (1073 (47.63%) men and 1180 (52.33%) women) with osteopenia were selected; the data analysis is given in Table 1. Americans who were female (*p* < 0.001), over 65 years old (*p* < 0.001), had a lower BMI (*p* < 0.001), were educated to high school graduation or equivalent or to less than 9^th^ grade (*p* = 0.049), or who had been born abroad (*p* = 0.008) were more likely to have an increased risk of osteopenia. A recent study [25] demonstrated that fracture incidence varies with socioeconomic status, race, and ethnicity, but race, ethnicity (*p* = 0.801), family income (*p* = 0.175), and current smoking status (*p* = 0.089) have no significant effect on BMD in the US.

### 3.2. Associations between Different Environmental Chemical Factors and Osteopenia

Means, SDs, and reference ranges of BMD and cut-off values of osteopenia and osteoporosis corresponding to the World Health Organization diagnostic criteria at major anatomic sites for the reference groups were classified by sex, race, and ethnicity [26]. The specific reference values are given in Table 2.

A multivariate logistic regression model was adjusted by covariates, demonstrating the association between environmental chemical factors and BMD at different skeletal sites (Table 3). For total body, lower levels exposure of urinary mercury (odds ratio (OR), 0.567; CI, 0.357–0.900; *p* = 0.016, 2-methylhippuric acid (OR, 0.425; CI, 0.232–0.778; *p* = 0.006), and N-acetyl-S-(2-hydroxypropyl)-L-cysteine (OR, 0.415; CI, 0.175–0.982; *p* = 0.045) were in statistically significant association with osteopenia. For the specific body sites, lower levels of urinary barium (OR, 0.130; CI, 0.017–0.983, *p* = 0.048) are obviously associated with left arm osteopenia; lower levels of N-acetyl-S-(n-propyl)-L-cysteine (OR, 0.502; CI, 0.298–0.844, *p* = 0.009) and N-acetyl-S-(phenyl-2-hydroxyethyl)-L-cysteine (OR, 0.552; CI, 0.324–0.939, *p* = 0.028) remained in statistically significant association with osteopenia in the right arm. Lower levels of urinary barium (OR, 0.088; CI, 0.009–0.864; *p* = 0.037), mercury (OR, 0.514; CI, 0.341–0.773; *p* = 0.001), 2-methylhippuric acid (OR, 0.487; CI, 0.275–0.863; *p* = 0.014), and N-acetyl-S-(2-hydroxypropyl)-L-cysteine (CYHA) (OR, 0.56; CI, 0.322–0.975; *p* = 0.04) remained in statistically significant association with left leg osteopenia. Lower levels of urinary barium (OR, 0.093; CI, 0.009–0.912; *p* = 0.041), mercury (OR, 0.529; CI, 0.350–0.798; *p* = 0.002), 2-methylhippuric acid (OR, 0.450; CI, 0.255–0.795; *p* = 0.006), and N-acetyl-S-(4-hydroxy-2-methyl-2-buten-1-yl)-L-cysteine (IPM3) (OR, 0.566; CI, 0.337–0.949, *p* = 0.031) were in statistically significant association with right leg osteopenia.

There is a strong association of high urinary manganese with osteopenia in the left arm (OR, 1.610; CI, 1.015–2.554; *p* = 0.043) or pelvis (OR, 1.587; CI, 1.001–2.516; *p* = 0.05) and monomethylarsonic acid (OR, 1.451; CI, 1.007–2.090; *p* = 0.046) with osteopenia of the lumbar spine. The results are given in Table 3.

### 3.3. Associations between Different Environmental Chemical Factors and Fracture

In this study, fracture data from the hip, wrist, and spine were combined into one dataset, owing to the small amount of data. Table 4 summarizes the associations of different environmental chemical factors with bone fractures at the hip, wrist, and spine in the multivariate models. Adjusted by covariants, high levels of arsenous acid (OR, 2.578, CI: 1.358–4.893; *p* = 0.004), arsenobetaine (OR, 2.978; CI, 1.052–8.427; *p* = 0.04), dimethylarsinic acid (OR, 2.087; CI, 1.06–4.112; *p* = 0.033), monomethylarsonic acid (OR, 2.276; CI, 1.225–4.226; *p* = 0.009), and 2-thioxothiazolidine-4-carboxylic acid (OR, 1.804; CI, 1.004–3.242; *p* = 0.048) were associated with an increased prevalence of osteoporotic fractures.

## 4. Discussion

Environmental chemical factors have different effects on bone development, and vary depending on the degree of exposure. In this cross-sectional study, we examined the association of environmental chemicals with BMD and bone fractures among a large sample of American adults from NHANES 2017–2018. The results in the present study demonstrate that urinary manganese, barium, mercury, dimethylarsinic acid, 2-methylhippuric acid, CYHA cysteine, N-acetyl-S-(2-hydroxypropyl)-L-cysteine, and IPM3 cysteine has a potential effect on osteopenia in American adults while not associated with fractures. Interestingly, the environmental chemicals (arsenous acid, arsenocholine, dimethylarsinic acid, monomethylarsonic acid, and 2-thioxothiazolidine-4-carboxylic acid) causing fracture are entirely different from the environmental chemicals that significantly reduce bone mass. The impact of environmental chemicals on fracture may not be caused by low bone mass. It is well known that osteoporosis is due to changes in bone microarchitecture and bone strength leading to fractures [27]. Bone strength is related to both BMD and bone microstructure [27]. However, only the assessment of BMD is available in clinical routine care today. Measuring bone turnover markers is the best non-invasive and easy non-time-consuming way to assess bone quality and the relationship between bone formation and bone resorption [28]. The latter increases physiologically with age, particularly in postmenopausal women, and may increase the risk of fractures.

Due to estrogen changes, fracture incidence in women over 50 is approximately twice that in men [29,30]; it is consistent with our findings. However, there is no information on the number of peri- and postmenopausal women in this database of 2017–2018 NHANES; it would be beneficial to investigate these data since the frequency of osteopenia in females was significantly higher than in males.

Previous data confirmed that environmental factors have an impact on BMD. Cadmium exposure inhibits osteoblast and osteoclast differentiation via the P2X7/PI3K/AKT signaling pathway, and causes osteoporosis by promoting osteoclast and osteoblast apoptosis [31]. In a steady state, bone contains about 80%–90% of the total body lead burden [32]. Cui et al. [33] showed that lead affected the lumbar BMD more than the femur, and that the impact on women was more robust. As mentioned above, the dietary intake of manganese is positively related to the lumbar spine, although one study found that blood manganese exposure was negatively related to BMD [34]. Based on these two results, our study found a strong positive correlation between urinary manganese exposure and osteopenia; this result is consistent with the study of Karamati et al. [12]. In addition, the zinc/copper ratio in serum and bone is positively correlated with systemic BMD and BMC in older men with osteoarthritis [35]. Serum selenium level was positively correlated with BMD in the forearm and heel [36]. However, dietary selenium intake as a function of BMD shows an inverted U-shaped trend, owing to the status of selenium in the body [37]. Blood mercury is positively associated with spine BMD and negatively associated with femoral BMD [38]. Our study confirms that urinary mercury is positively associated with the BMD of the legs and total body. Furthermore, to our knowledge, we did not find any research on the relationship between barium and BMD; however, we found that low-level urinary barium exposure in the left arm and legs may significantly increase the risk of osteopenia.

Nguyen et al. [39] indicated that PAHs could inhibit absorption of osteoclasts and synthesis of osteoblasts, and damage bone remodeling. Izawa et al. [40] suggested that benzopyrene accelerated the development of osteoclasts. High exposure to 3-methylcholanthrene [41] results in bone loss and decreases bone mechanical properties. Hsueh et al. [42] demonstrated that high total urinary arsenic levels significantly increased the risk of bone loss. Consistently, we found that high urinary levels of monomethylarsonic acid had a significant correlation with osteopenia, and that high levels of arsenous acid, arsenocholine, dimethylarsinic acid, and dimethylarsinic acid significantly increased the possibility of fragility fracture. No other environmental factors (including heavy metals) have a significant impact on fracture. Subsequently, our study verified a negative correlation between lower VOC exposure with osteopenia. Some factors, including 2-methylhippuric acid, N-acetyl-S-(2-hydroxypropyl)-L-cysteine, N-acetyl-S-(phenyl-2-hydroxyethyl)-L-cysteine, CYHA cysteine, and IPM3 cysteine, and 2-thioxothiazolidine-4-carboxylic acid, have a potential role in osteopenia and only 2-Thioxothiazolidine-4-carboxylic acid was found to increase the risk of fracture significantly.

This study has some limitations. First, the sample size is not large enough to obtain more results specific to each bone region, and due to the lack of fracture data for the arm and pelvis, we cannot determine whether manganese increases the risk of fracture, although manganese has a significant effect on BMD reduction. More data should be collected to investigate the effects of environmental chemical factors on different bone regions. Moreover, the data of the individuals chosen in this study, collected in 2017 and 2018, can only reflect a short-term influence of environmental chemical factors; the time span should be extended for long-term effects.

## 5. Conclusions

The results of this study demonstrate that environmental chemical factors have strong associations with osteopenia and/or fracture. However, several factors (especially arsenic compounds) cause bone fragility fracture, but no bone loss. We considered that environmental chemical-induced fracture is independent of BMD. Further studies should explore the exact mechanisms of these factors, which will be helpful in the prevention and treatment of bone diseases caused by environmental problems.

## Figures and Tables

**Table 1:** Characteristics of American adults with osteopenia in 2017–2018.

*n* = 2253	No. Reported	No Osteopenia (%)	Osteopenia (%)	*p* Value
**Age**	2253	81.4	18.6	<0.001
**Gender**	2253			
Male	1073	84.7	15.3	<0.001
Female	1180	78.3	21.7	
**Body mass index**	2253			
<18.5	43	51.2	48.8	<0.001
18.5–24.9	625	75.8	24.2	
25–29.9	699	84.0	16.0	
30+	876	84.6	15.4	
**Race and ethnicity**	2253			
Mexican American	350	82.6	17.4	0.801
Non-Hispanic White	684	82.0	18.0	
Non-Hispanic Black	445	79.6	20.4	
Non-Hispanic Asian	416	80.8	19.2	
Other Hispanic	225	79.1	20.9	
Other Race—Including Multi-Racial	133	86.5	15.5	
**Education level**	2252			
Less than 9th grade	132	78.0	22.0	0.049
Some high school	243	84.8	15.2	
High school graduate or equivalent	515	74.8	25.2	
Some college or AA degree	762	83.9	16.1	
College graduate or above	600	83.2	16.8	
**Born in USA**	2252			
Yes	1441	83.0	17.0	0.008
No	811	78.4	21.6	
**Ratio of family income to poverty**	2002			
0–4.9	1647	82.0	18.0	0.175
5+	355	78.9	21.1	
**Current** **smoker status**	824			
Every day	361	77.8	22.2	0.089
Some days	96	82.3	17.7	
Not at all	367	82.8	17.2	

**Table 2:** Mean bone mineral density of osteopenia and osteoporosis aged 20–29 years in 2017–2018.

	Male					Female				
	Mean (g/cm^2^)	Standard Deviation (g/cm^2^)	Range	Osteopenia	Osteoporosis	Mean (g/cm^2^)	Standard Deviation (g/cm^2^)	Range	Osteopenia	Osteoporosis
**Mexican American**										
Total body	1.138	0.084	0.987–1.482	0.928–1.054	<0.928	1.068	0.079	0.925–1.305	0.871–0.989	<0.871
Upper body										
Left arm	0.821	0.067	0.713–1.017	0.654–0.754	<0.654	0.688	0.048	0.597–0.804	0.568–0.640	<0.568
Right arm	0.838	0.065	0.712–1.067	0.676–0.773	<0.676	0.707	0.044	0.601–0.822	0.597–0.663	<0.597
Total thoracic spine	0.842	0.093	0.668–1.104	0.610–0.749	<0.610	0.796	0.080	0.573–1.011	0.596–0.716	<0.596
Lower body										
Left leg	1.240	0.090	1.032–1.527	1.015–1.15	<1.015	1.090	0.100	0.926–1.324	0.840–0.990	<0.840
Right leg	1.254	0.104	1.001–1.579	0.994–1.15	<0.994	1.092	0.091	0.956–1.296	0.865–1.001	<0.865
Total lumbar spine	1.003	0.120	0.801–1.339	0.703–0.883	<0.703	1.010	0.120	0.833–1.377	0.710–0.890	<0.710
Pelvis	1.278	0.133	1.035–1.787	0.946–1.145	<0.946	1.205	0.141	0.941–1.592	0.853–1.064	<0.853
**Non-Hispanic White**										
Total body	1.144	0.100	0.893–1.363	0.894–1.044	<0.894	1.095	0.084	0.940–1.348	0.885–1.011	<0.885
Upper body										
Left arm	0.840	0.081	0.659–1.108	0.638–0.759	<0.638	0.718	0.049	0.614–0.853	0.596–0.669	<0.596
Right arm	0.869	0.088	0.664–1.124	0.649–0.781	<0.649	0.730	0.049	0.635–0.846	0.608–0.681	<0.608
Total thoracic spine	0.835	0.101	0.536–1.110	0.583–0.734	<0.583	0.814	0.091	0.648–1.160	0.587–0.723	<0.587
Lower body										
Left leg	1.231	0.121	0.890–1.459	0.929–1.11	<0.929	1.124	0.085	0.949–1.347	0.912–1.039	<0.912
Right leg	1.238	0.117	0.928–1.456	0.946–1.121	<0.946	1.138	0.085	0.921–1.331	0.926–1.053	<0.926
Total lumbar spine	1.038	0.133	0.759–1.358	0.706–0.905	<0.706	1.069	0.126	0.831–1.556	0.754–0.943	<0.754
Pelvis	1.293	0.181	0.835–1.863	0.841–1.112	<0.841	1.243	0.135	0.973–1.605	0.906–1.108	<0.906
**Non-Hispanic Black**										
Total body	1.216	0.104	0.942–1.517	0.956–1.112	<0.956	1.136	0.067	0.960–1.271	0.969–1.069	<0.969
Upper body										
Left arm	0.854	0.068	0.709–1.074	0.684–0.786	<0.684	0.744	0.060	0.643–0.957	0.594–0.684	<0.594
Right arm	0.881	0.079	0.711–1.132	0.684–0.802	<0.684	0.761	0.066	0.667–0.997	0.596–0.695	<0.596
Total thoracic spine	0.884	0.108	0.642–1.164	0.614–0.776	<0.614	0.843	0.073	0.666–1.015	0.661–0.770	<0.661
Lower body										
Left leg	1.322	0.126	1.045–1.694	1.007–1.196	<1.007	1.158	0.089	0.964–1.378	0.936–1.069	<0.936
Right leg	1.333	0.121	1.043–1.602	1.031–1.212	<1.031	1.182	0.091	0.993–1.419	0.955–1.091	<0.955
Total lumbar spine	1.145	0.174	0.761–1.646	0.710–0.971	<0.710	1.105	0.122	0.886–1.406	0.800–0.983	<0.800
Pelvis	1.343	0.208	0.972–1.978	0.823–1.135	<0.823	1.288	0.157	0.988–1.637	0.896–1.131	<0.896
**Non-Hispanic Asian**										
Total body	1.115	0.100	0.918–1.302	0.865–1.015	<0.865	1.060	0.068	0.908–1.192	0.890–0.992	<0.890
Upper body										
Left arm	0.791	0.073	0.644–0.964	0.609–0.718	<0.609	0.663	0.042	0.560–0.739	0.558–0.621	<0.558
Right arm	0.819	0.065	0.686–0.938	0.657–0.754	<0.657	0.684	0.041	0.584–0.763	0.582–0.643	<0.582
Total thoracic spine	0.815	0.089	0.613–0.978	0.593–0.726	<0.593	0.777	0.085	0.613–0.992	0.565–0.692	<0.565
Lower body										
Left leg	1.214	0.109	0.952–1.489	0.942–1.105	<0.942	1.068	0.081	0.906–1.266	0.866–0.987	<0.866
Right leg	1.208	0.111	0.949–1.447	0.931–1.097	<0.931	1.084	0.080	0.929–1.249	0.884–1.004	<0.884
Total lumbar spine	1.053	0.148	0.758–1.437	0.683–0.905	<0.683	1.009	0.103	0.837–1.210	0.752–0.906	<0.752
Pelvis	1.260	0.167	0.901–1.620	0.843–1.093	<0.843	1.181	0.131	0.979–1.476	0.854–1.050	<0.854
**Other Hispanic**										
Total body	1.149	0.090	0.940–1.323	0.924–1.059	<0.924	1.065	0.061	0.919–1.181	0.913–1.004	<0.913
Upper body										
Left arm	0.835	0.069	0.732–1.044	0.663–0.766	<0.663	0.699	0.051	0.637–0.687	0.572–0.648	<0.572
Right arm	0.851	0.054	0.759–0.979	0.716–0.797	<0.716	0.716	0.040	0.646–0.823	0.616–0.676	<0.616
Total thoracic spine	0.848	0.100	0.664–1.042	0.598–0.748	<0.598	0.803	0.077	0.670–0.975	0.611–0.726	<0.611
Lower body										
Left leg	1.225	0.106	1.006–1.481	0.96–1.119	<0.96	1.102	0.082	0.963–1.345	0.897–1.020	<0.897
Right leg	1.223	0.095	1.026–1.434	0.986–1.128	<0.986	1.111	0.091	0.947–1.368	0.884–1.020	<0.884
Total lumbar spine	1.028	0.130	0.842–1.432	0.703–0.898	<0.703	1.030	0.137	0.827–1.433	0.688–0.893	<0.688
Pelvis	1.316	0.186	1.040–1.939	0.851–1.130	<0.851	1.200	0.110	1.046–1.542	0.925–1.090	<0.925
**Other race**										
Total body	1.187	0.118	0.953–1.362	0.892–1.069	<0.892	1.082	0.092	0.911–1.251	0.852–0.990	<0.852
Upper body										
Left arm	0.875	0.100	0.720–1.040	0.625–0.775	<0.625	0.699	0.063	0.616–0.866	0.542–0.636	<0.542
Right arm	0.911	0.116	0.736–1.154	0.621–0.795	<0.621	0.723	0.077	0.616–0.898	0.531–0.646	<0.531
Total thoracic spine	0.874	0.098	0.656–1.011	0.629–0.776	<0.629	0.818	0.120	0.628–1.155	0.518–0.698	<0.518
Lower body										
Left leg	1.297	0.119	1.096–1.549	1.000–1.178	<1.000	1.098	0.090	0.987–1.334	0.873–1.008	<0.873
Right leg	1.305	0.160	1.020–1.630	0.905–1.145	<0.905	1.110	0.106	0.992–1.391	0.845–1.004	<0.845
Total lumbar spine	1.073	0.151	0.849–1.386	0.696–0.922	<0.696	1.068	0.117	0.903–1.284	0.776–0.951	<0.776
Pelvis	1.359	0.165	1.070–1.699	0.947–1.194	<0.947	1.245	0.125	1.052–1.496	0.933–1.120	<0.933

WHO diagnostic criteria: osteopenia, BMD value between 1 and 2.5 SD below mean of reference group; osteoporosis, BMD value 2.5 SD below mean of reference group.

**Table 3:** Associations with multiple environmental chemical factors and osteopenia in 2017–2018.

*n* = 2640	Total Body		Left Arm		Right Arm		Total Thoracic Spine	
	OR (95%CI)	*p* Value	OR (95%CI)	*p* Value	OR (95%CI)	*p* Value	OR (95%CI)	*p* Value
**Metals(ug/L) ^a^**								
Barium	0.277 (0.023–3.330)	0.312	**0.130 (0.017–0.983)**	**0.048**	0.297 (0.025–3.559)	0.338	0.459 (0.440–4.418)	0.517
Cadmiu	0.862 (0.330–2.251)	0.761	0.636 (0.273–1.483)	0.295	1.610 (0.538–4.815)	0.395	0.973 (0.395–2.398)	0.953
Manganese	1.428 (0.904–2.254)	0.127	**1.610 (1.015–2.554)**	**0.043**	1.327 (0.838–2.102)	0.227	1.235 (0.770–1.981)	0.381
Antimony	1.157 (0.671–1.994)	0.6	0.764 (0.456–1.280)	0.306	0.733 (0.441–1.216)	0.228	1.064 (0.615–1.84)	0.825
Tin	0.540 (0.278–1.047)	0.068	0.532 (0.275–1.03)	0.061	0.707 (0.355–1.405	0.322	0.965 (0.479–1.944)	0.922
Tungsten	0.711 (0.395–1.279)	0.255	0.831 (0.453–1.527)	0.552	0.778 (0.432–1.401)	0.403	0.886 (0.477–1.646)	0.701
Chromium	0.965 (0.599–1.554)	0.882	0.980 (0.605–1.588)	0.935	1.192 (0.748–1.899)	0.459	0.898 (0.552–1.462)	0.666
Mercury	**0.567 (0.357–0.900)**	**0.016**	0.741 (0.467–1.177)	0.204	0.732 (0.463–1.157)	0.182	1.114 (0.699–1.774)	0.651
**Arsenic(ug/L) ^a^**								
Arsenous acid	1.027 (0.648–1.629)	0.91	1.036 (0.649–1.652)	0.883	1.182 (0.748–1.868)	0.473	1.050 (0.662–1.665)	0.836
Arsenic acid	0.707 (0.262–1.908)	0.494	0.610 (0.210–1.771)	0.363	0.513 (0.176–1.497)	0.222	0.452 (0.133–1.543)	0.205
Arsenobetaine	1.171 (0.737–1.860)	0.505	0.696 (0.431–1.125)	0.139	0.681 (0.424–1.092)	0.111	1.107 (0.694–1.766)	0.67
Arsenocholine	0.603 (0.256–1.418)	0.246	0.629 (0.257–1.542)	0.311	0.408 (0.155–1.071)	0.069	0.729 (0.312–1.706)	0.467
Dimethylarsinic acid	0.923 (0.562–1.515)	0.751	0.621 (0.383–1.009)	0.054	0.686 (0.422–1.115)	0.129	1.119 (0.668–1.874)	0.669
Monomethylarsonic acid	0.781 (0.496–1.231)	0.288	0.916 (0.579–1.449)	0.707	1.243 (0.789–1.957)	0.348	0.885 (0.561–1.397)	0.6
**Volatile Organic Compound (VOC) Metabolites(ng/mL) ^a^**								
2-methylhippuric acid	**0.425 (0.232–0.778)**	**0.006**	0.826 (0.430–1.584)	0.564	0.661 (0.353–1.237)	0.195	0.927 (0.473–1.814)	0.824
3-methipurc acid and 4-methipurc acid	0.465 (0.105–2.060)	0.313	0.838 (0.163–4.316)	0.833	0.910 (0.178–4.646)	0.91	0.680 (0.119–3.877)	0.664
2-amnothiazolne-4-carbxylic acid	0.801 (0.424–1.514)	0.495	0.579 (0.315–1.063)	0.078	0.686 (0.370–1.274)	0.233	1.062 (0.536–2.102)	0.864
N-acetyl-S-(n-propyl)-L-cysteine	0.891 (0.512–1.548)	0.681	0.618 (0.361–1.056)	0.078	**0.502 (0.298–0.844)**	**0.009**	0.908 (0.512–1.612)	0.742
N-acetyl-S-(2-carbxyethyl)-L-cys	0.890 (0.166–4.779)	0.892	1.818 (0.215–15.388)	0.584	1.290 (0.245–6.798)	0.764	1.826 (0.218–15.305)	0.579
CYHA cysteine	0.858 (0.464–1.587)	0.625	1.051 (0.569–1.942)	0.874	0.811 (0.430–1.529)	0.517	0.925 (0.504–1.699)	0.803
N-acetyl-S-(2-cyanoethyl)-L-cyst	0.875 (0.503–1.520)	0.635	0.979 (0.553–1.733)	0.942	0.759 (0.444–1.299)	0.315	1.441 (0.762–2.724)	0.261
N-ac-S-(2-carbmo-2-hydxel)-L-cys	1.055 (0.659–1.690)	0.824	0.933 (0.579–1.503)	0.774	1.005 (0.625–1.616)	0.984	0.739 (0.454–1.202)	0.223
N-ace-S-(2-hydroxyethyl)-L-cys	1.004 (0.631–1.598)	0.987	0.683 (0.421–1.108)	0.122	0.685 (0.424–1.107)	0.123	0.979 (0.611–1.570)	0.931
N-ace-S-(2-hydroxypropyl)-L-cys	**0.415 (0.175–0.982)**	**0.045**	0.652 (0.274–1.554)	0.334	0.770 (0.309–1.919)	0.575	0.995 (0.372–2.659)	0.992
N-ace-S-(3-hydroxypropyl)-L-cys	0.101 (0.008–1.232)	0.072	0.097 (0.009–1.112)	0.061	0.105 (0.009–1.202)	0.07	0.088 (0.007–1.025)	0.052
IPM3 cysteine	0.648 (0.368–1.138)	0.131	0.961 (0.531–1.741)	0.896	0.793 (0.448–1.402)	0.425	0.701 (0.397–1.239)	0.222
Mandelic acid	0.292 (0.065–1.311)	0.108	0.444 (0.086–2.286)	0.332	0.280 (0.063–1.241)	0.094	0.350 (0.064–1.904)	0.225
N-A-S-(4-hydroxy-2-butenyl)-L-cys	0.560 (0.198–1.582)	0.274	0.699 (0.241–2.028)	0.509	0.612 (0.223–1.683)	0.342	0.690 (0.226–2.108)	0.515
N-ace-S-(phenl-2-hydxyetl)-L-cys	0.848 (0.517–1.391)	0.514	0.753 (0.452–1.255)	0.277	**0.552 (0.324–0.939)**	**0.028**	0.870 (0.528–1.432)	0.583
2-Thioxothiazolidine-4-carboxylic acid	1.013 (0.638–1.608)	0.955	0.890 (0.553–1.432)	0.63	0.718 (0.443–1.164)	0.179	1.165 (0.729–1.862)	0.524
**Metals (ug/L) ^a^**								
Barium	**0.088 (0.009–0.864)**	**0.037**	**0.093 (0.009–0.912)**	**0.041**	0.238 (0.33–1.736)	0.157	NA	NA
Cadmiu	0.975 (0.415–2.291)	0.954	0.851 (0.371–1.954)	0.704	1.005 (0.452–2.233)	0.99	0.62 (0.261–1.47)	0.278
Manganese	1.372 (0.915–2.057)	0.126	1.310 (0.869–1.974)	0.198	1.367 (0.946–1.974)	0.096	**1.587 (1.001–2.516)**	**0.05**
Antimony	0.767 (0.484–1.215)	0.258	0.860 (0.538–1.374)	0.528	0.832 (0.54–1.28)	0.402	1.246 (0.704–2.205)	0.451
Tin	0.673 (0.363–1.248)	0.209	0.731 (0.395–1.353)	0.319	0.999 (0.543–1.837)	0.998	0.667 (0.35–1.274)	0.22
Tungsten	1.222 (0.695–2.149)	0.486	1.181 (0.676–2.062)	0.558	0.829 (0.498–1.379)	0.47	1.146 (0.605–2.168)	0.676
Chromium, Urine	0.829 (0.544–1.263)	0.382	0.836 (0.547–1.278)	0.408	1.048 (0.72–1.526)	0.805	0.925 (0.574–1.492)	0.75
Mercury	**0.514 (0.341–0.773)**	**0.001**	**0.529 (0.350–0.798)**	**0.002**	1.02 (0.709–1.466)	0.916	0.995 (0.627–1.581)	0.984
**Arsenic (ug/L) ^a^**								
Arsenous acid	0.893 (0.595–1.340)	0.584	0.872 (0.580–1.311)	0.51	1.235 (0.858–1.778)	0.256	0.847 (0.533–1.347)	0.484
Arsenic acid	0.631 (0.267–1.491)	0.294	0.745 (0.328–1.694)	0.483	0.942 (0.424–2.093)	0.884	0.252 (0.059–1.084)	0.064
Arsenobetaine	0.841 (0.559–1.264)	0.405	0.705 (0.465–1.070)	0.101	1.081 (0.753–1.553)	0.672	1.057 (0.665–1.681)	0.815
Arsenocholine	0.924 (0.468–1.825)	0.82	0.728 (0.355–1.496)	0.388	1.07 (0.582–1.968)	0.827	0.668 (0.286–1.559)	0.351
Dimethylarsinic acid	**0.615 (0.401–0.942)**	**0.025**	0.710 (0.462–1.091)	0.118	1.153 (0.755–1.759)	0.51	0.858 (0.521–1.414)	0.549
Monomethylarsonic acid	0.828 (0.557–1.231)	0.35	0.801 (0.538–1.192)	0.273	**1.451 (1.007–2.090)**	**0.046**	1.075 (0.682–1.693)	0.756
**Volatile Organic Compound (VOC) Metabolites (ng/mL) ^a^**								
2-methylhippuric acid	**0.487 (0.275–0.863)**	**0.014**	**0.450 (0.255–0.795)**	**0.006**	0.793 (0.456–1.381)	0.413	0.686 (0.347–1.356)	0.278
3-methipurc acid and 4-methipurc acid	1.181 (0.220–6.332)	0.846	1.159 (0.210–6.382)	0.865	0.453 (0.106–1.934)	0.285	1.526 (0.175–13.307)	0.702
2-amnothiazolne-4-carbxylic acid	0.724 (0.414–1.267)	0.258	0.886 (0.499–1.575)	0.681	1.231 (0.701–2.163)	0.469	1.216 (0.617–2.397)	0.573
N-acetyl-S-(n-propyl)-L-cysteine	0.802 (0.491–1.307)	0.376	0.781 (0.482–1.267)	0.318	0.378 (0.757–2.080)	0.378	0.884 (0.501–1.562)	0.672
N-acetyl-S-(2-carbxyethyl)-L-cys	2.981 (0.347–25.651)	0.32	1.003 (0.189–5.323)	0.998	1.733 (0.21–14.303)	0.61	0.337 (0.074–1.544)	0.161
CYHA cysteine	**0.56 (0.322–0.975)**	**0.04**	0.705 (0.413–1.202)	0.199	0.727 (0.468–1.129)	0.156	1.145 (0.647–2.027)	0.641
N-acetyl-S-(2-cyanoethyl)-L-cyst	0.690 (0.422–1.128)	0.139	0.768 (0.469–1.259)	0.295	1.013 (0.618–1.662)	0.959	0.912 (0.502–1.657)	0.763
N-ac-S-(2-carbmo-2-hydxel)-L-cys	0.804 (0.532–1.215)	0.3	0.826 (0.546–1.250)	0.366	0.827 (0.572–1.196)	0.312	1.133 (0.708–1.811)	0.603
N-ace-S-(2-hydroxyethyl)-L-cys	0.845 (0.560–1.275)	0.422	0.810 (0.534–1.229)	0.322	0.935 (0.647–1.351)	0.72	1.109 (0.694–1.773)	0.666
N-ace-S-(2-hydroxypropyl)-L-cys	0.559 (0.246–1.268)	0.164	0.519 (0.233–1.157)	0.109	0.656 (0.296–1.456)	0.3	0.77 (0.288–2.060)	0.602
N-ace-S-(3-hydroxypropyl)-L-cys	0.154 (0.014–1.748)	0.131	0.157 (0.014–1.798)	0.137	0.115 (0.01–1.323)	0.083	0.274 (0.023–3.201)	0.302
IPM3 cysteine	0.642 (0.384–1.072)	0.09	**0.566 (0.337–0.949)**	**0.031**	0.802 (0.5–1.285)	0.358	0.951 (0.516–1.753)	0.873
Mandelic acid	0.764 (0.147–3.966)	0.749	0.709 (0.137–3.674)	0.682	0.627 (0.119–3.308)	0.582	0.932 (0.109–8.066)	0.949
N-A-S-(4-hydroxy-2-butenyl)-L-cys	0.735 (0.260–2.074)	0.561	0.562 (0.205–1.543)	0.263	0.909 (0.33–2.502)	0.853	0.526 (0.173–1.597)	0.257
N-ace-S-(phenl-2-hydxyetl)-L-cys	0.725 (0.469–1.120)	0.148	0.809 (0.526–1.246)	0.337	0.725 (0.493–1.067)	0.103	0.682 (0.413–1.128)	0.136
2-Thioxothiazolidine-4-carboxylic acid	1.094 (0.730–1.640)	0.664	1.049 (0.696–1.579)	0.821	0.99 (0.684–1.431)	0.956	1.151 (0.725–1.826)	0.552

^a^ Covariates adjusted for age, gender, body mass index, race and ethnicity, education level, country of birth, ratio of family income to poverty, and current smoker status.

**Table 4:** Associations with multiple environmental chemical factors and fractures in 2017–2018.

*n* = 292	No Fracture	Fracture	OR (95%CI)	*p* Value
**Metals (ug/L) ^a^**				
Cadmium	0.489 ± 0.585	0.442 ± 0.436	1.488 (0.27–8.218)	0.648
Manganese	0.174 ± 0.217	0.286 ± 1.194	1.11 (0.611–2.015)	0.732
Antimony	0.074 ± 0.199	0.095 ± 0.132	1.163 (0.539–2.508)	0.701
Tin	1.013 ± 2.466	1.051 ± 1.590	5.016 (0.589–42.727)	0.14
Tungsten	0.191 ± 1.171	0.076 ± 0.055	2.402 (0.832–6.938)	0.105
Chromium	0.294 ± 0.436	0.436 ± 1.316	1.249 (0.692–2.253)	0.461
Mercury	0.425 ± 0.883	0.407 ± 0.561	1.348 (0.741–2.453)	0.329
**Arsenic (ug/L) ^a^**				
Arsenous acid	0.274 ± 0.394	0.304 ± 0.373	**2.578 (1.358–4.893)**	**0.004**
Arsenic acid	0.595 ± 0.220	0.612 ± 0.258	1.468 (0.495–4.352)	0.488
Arsenobetaine	8.736 ± 35.66	9.121 ± 30.560	1.419 (0.787–2.558)	0.244
Arsenocholine	0.147 ± 0.503	0.114 ± 0.127	**2.978 (1.052–8.427)**	**0.04**
Dimethylarsinic acid	6.278 ± 11.100	4.769 ± 5.409	**2.087 (1.06–4.112)**	**0.033**
Monomethylarsonic acid	0.505 ± 0.655	0.570 ± 0.624	**2.276 (1.225–4.226)**	**0.009**
**Volatile Organic Compound (VOC) Metabolites (ng/mL) ^a^**				
2-methylhippuric acid	62.82 ± 109.600	158.700 ± 807.300	3.532 (0.957–13.031)	0.058
2-amnothiazolne-4-carbxylic acid	181.900 ± 295.400	177.900 ± 250.900	0.822 (0.372–1.816)	0.628
N-acetyl-S-(n-propyl)-L-cysteine	10.450 ± 17.470	11.580 ± 14.390	1.155 (0.546–2.445)	0.705
N-acetyl-S-(2-carbxyethyl)-L-cys	171.800 ± 211.100	216.400 ± 234.100	0.713 (0.052–9.848)	0.8
CYHA cysteine	10.330 ± 26.510	14.990 ± 29.750	1.378 (0.664–2.863)	0.389
N-acetyl-S-(2-cyanoethyl)-L-cyst	51.360 ± 118.900	69.880 ± 137.600	1.933 (0.859–4.348)	0.111
N-ac-S-(2-carbmo-2-hydxel)-L-cys	12.790 ± 13.340	14.870 ± 14.030	1.678 (0.925–3.043)	0.088
N-ace-S-(2-hydroxyethyl)-L-cys	1.836 ± 4.934	1.630 ± 2.178	1.345 (0.739–2.451)	0.332
N-ace-S-(2-hydroxypropyl)-L-cys	57.68 ± 120.900	72.39 ± 113.3	2.245 (0.436–11.560)	0.333
N-ace-S-(3-hydroxypropyl)-L-cys	540.200 ± 996.600	763.200 ± 1111.000	0.592 (0.035–9.870)	0.715
IPM3 cysteine	18.400 ± 37.970	23.800 ± 42.800	1.28 (0.523–3.133)	0.588
Mandelic acid	212.300 ± 284.800	284.700 ± 370.900	0.592 (0.035–9.870)	0.715
N-A-S-(4-hydroxy-2-butenyl)-L-cys	14.140 ± 25.480	16.580 ± 26.190	1.492 (0.141–15.749)	0.739
N-ace-S-(phenl-2-hydxyetl)-L-cys	1.477 ± 2.032	1.938 ± 3.581	1.413 (0.749–2.668)	0.286
2-Thioxothiazolidine-4-carboxylic acid	40.010 ± 109.800	29.780 ± 46.210	**1.804 (1.004–3.242)**	**0.048**

^a^ Covariates adjusted for age, gender, body mass index, race and ethnicity, education level, country of birth, ratio of family income to poverty, and current smoker status.

## Data Availability

Data available in a publicly accessible repository. Publicly available datasets were analyzed in this study. This data can be found in the National Health and Nutrition Examination Survey, https://wwwn.cdc.gov/nchs/nhanes/Default.aspx (accessed on 30 January 2023).
